# The effect of schizophrenia risk factors on mismatch responses in a rat model

**DOI:** 10.1111/psyp.14175

**Published:** 2022-09-10

**Authors:** Jaishree Jalewa, Juanita Todd, Patricia T. Michie, Deborah M. Hodgson, Lauren Harms

**Affiliations:** ^1^ School of Psychological Sciences, College of Engineering, Science and Environment University of Newcastle Callaghan New South Wales Australia; ^2^ Hunter Medical Research Institute New Lambton Heights New South Wales Australia; ^3^ School of Biomedical Science and Pharmacy, College of Health, Medicine and Wellbeing University of Newcastle Callaghan New South Wales Australia

**Keywords:** cannabis, context, deviance difference, electroencephalography, maternal immune activation, mismatch negativity, probability, rat

## Abstract

Reduced mismatch negativity (MMN), a robust finding in schizophrenia, has prompted interest in MMN as a preclinical biomarker of schizophrenia. The rat brain can generate human‐like mismatch responses (MMRs) which therefore enables the exploration of the neurobiology of reduced MMRs. Given epidemiological evidence that two developmental factors, maternal infection and adolescent cannabis use, increase the risk of schizophrenia, we determined the effect of these two developmental risk factors on rat MMR amplitude in different auditory contexts. MMRs were assessed in awake adult male and female Wistar rats that were offspring of pregnant dams treated with either a viral infection mimetic (poly I:C) inducing maternal immune activation (MIA) or saline control. In adolescence, subgroups of the prenatal treatment groups were exposed to either a synthetic cannabinoid (adolescent cannabinoid exposure: ACE) or vehicle. The context under which MMRs were obtained was manipulated by employing two different oddball paradigms, one that manipulated the physical difference between rare and common auditory stimuli, and another that manipulated the probability of the rare stimulus. The design of the multiple stimulus sequences across the two paradigms also allowed an investigation of context on MMRs to two identical stimulus sequences. Male offspring exposed to each of the risk factors for schizophrenia (MIA, ACE or both) showed a reduction in MMR, which was evident only in the probability paradigm, with no effects seen in the physical difference. Our findings highlight the importance of contextual factors induced by paradigm manipulations and sex for modeling schizophrenia‐like MMN impairments in rats.

## Abstract 2

Our findings demonstrate a schizophrenia‐like reduction in the size of the mismatch responses in a developmental risk factor rat model, a novel finding as the previous research have shown similar effects in pharmacologically manipulated animal models only. The current results underscore the importance of paradigm in utilizing the mismatch negativity as a biomarker for schizophrenia in future studies.

## 1 INTRODUCTION

Affecting about 1% of the population worldwide, schizophrenia is a severe, chronic and debilitating mental illness caused by both genetic and environmental factors. In addition to the distress caused by the psychotic symptoms of schizophrenia, cognitive deficits impede the ability of patients to perform everyday tasks and are a major cause of poor functional outcome, thus adding to the social and economic burden of the disorder (Featherstone et al., 2018; Gault et al., 2018; Green, 2006; Hochberger et al., 2020; Javitt & Sweet, 2015; Lesh et al., 2011; Spellman & Gordon, 2015). The cognitive deficits seem to have somewhat separate neurobiological underpinnings compared to psychotic symptoms, as currently available antipsychotics do little for cognitive impairments. Animal models are an important tool for investigating the neural mechanisms underlying the cognitive impairments of schizophrenia, as well as for screening new treatments for the disorder. There are many different approaches for assessing the face validity of schizophrenia animal models for behavioral and molecular outcome measures (Forrest et al., 2014; Jones et al., 2011), however, electrophysiological outcomes, measured by the electroencephalogram (EEG) are somewhat unique due to the ability to measure very similar neurophysiological features in both animals and humans (O'Donnell, 2013).

Event‐related potentials (ERPs) are averaged electrophysiological responses to a stimulus, and represent sensory and cognitive processing of stimulus information (Rissling et al., 2010). Mismatch negativity (MMN), an ERP component that is induced in response to an unexpected event, is considered a promising biomarker for schizophrenia due to the robust findings of MMN amplitude reduction in more than 100 studies (Bodatsch et al., 2015; Erickson et al., 2016; Shelley et al., 1991; Umbricht & Krljes, 2005) of chronic, as well as recent‐onset schizophrenia patients (Avissar & Javitt, 2018; Light & Swerdlow, 2015; Nagai et al., 2013). MMN is automatically elicited in response to a rare, unexpected *deviant* stimulus (DEV) among expected common *standard* stimuli (STD) (Garrido et al., 2009; Näätänen & Alho, 1995). MMN amplitude is observed as a difference waveform (DEV‐STD) at approximately 100–200 ms post‐deviance (Javitt et al., 2008; Näätänen et al., 1978; Näätänen & Alho, 1995). Javitt et al. (1998) have shown that the deficit in MMN generation in schizophrenia varies as a function of stimulus conditions, which suggests that not only is MMN reduced in schizophrenia, but the way MMN size changes with stimulus conditions is disrupted, due most likely to a decrease in the maximal MMN amplitude that can be generated within the cortex of schizophrenia patients.

Various theoretical accounts of MMN have been proposed. Early hypotheses posed two interpretations for MMN – (i) a memory based hypothesis in which sensory memory of acoustic regularities induced by repetition of standards supports the detection of sensory deviance between the memory and sensory input (Näätänen et al., 2001) and (ii) an adaptation hypothesis which asserts that repeated presentations of the same sound stimulus produce attenuated exogenous ERP responses relative to responses to rare deviants (Jääskeläinen et al., 2004) (but see Näätänen et al., 2005 for a rebuttal of this latter view). Current theoretical accounts of MMN incorporate MMN generation into a predictive coding framework, a more general theory of brain function underpinning perceptual inference and learning, which incorporates aspects of the memory based hypothesis and adaptation (Garrido et al., 2009). Predictive coding proposes that perception is the result of the integration of sensory input with predictions about the likely characteristics of input based on prior exposure. MMN is considered to be the neural signature of a larger ‘prediction error’ generated when there is a discrepancy between prediction and sensory input, such as the occurrence of an unexpected deviant sound (Näätänen et al., 2001). Major determinants of prediction error reflected in the amplitude of MMN include the degree of precision in the predictive model determined by stability (less variability) of background regularities (Yon & Frith, 2021), and how far the deviant falls from the predicted parameters (Lieder et al., 2013). Hence MMN amplitude indexes precision‐weighting on the prediction error signal. Prediction error can trigger an update to the existing predictive model in order to minimize errors of prediction, a process believed to be implemented within a perceptual inference network including frontal and temporal brain regions (Garrido et al., 2009) and is dependent on N‐methyl‐D‐aspartate (NMDA) mediated synaptic plasticity (Friston, 2005; Garrido et al., 2009). Consistent with this interpretation, NMDA receptors (NMDAr) are implicated in MMN generation as NMDAr antagonists reduce MMN amplitude in healthy individuals (Umbricht et al., 2000, 2002). Methodologies using innovative sound sequences have been developed that demonstrate a substantial contribution of prediction error to MMN in humans that is independent of adaptation processes (Jacobsen & Schroger, 2001, 2003). Furthermore, it has been recently demonstrated that it is the adaptation‐independent prediction error component of MMN that is reduced in patients with schizophrenia (Koshiyama et al., 2020).

The ability to measure and manipulate human‐like MMN (mismatch responses, MMRs) in rats provides us with an opportunity to understand the underlying neurobiology of MMN and to develop a translational tool for validation of new pre‐clinical schizophrenia models based on this neurophysiological feature (Harms et al., 2016). Previously, our group and others have demonstrated that rat MMRs mimic attributes of human MMN, such as adaptation independence (Astikainen et al., 2011; Harms et al., 2014; Nakamura et al., 2011; Polterovich et al., 2018) and amplitude reduction associated with pharmacological NMDAr antagonism (Harms et al., 2018; Tikhonravov et al., 2008). Our findings suggest that a negative component in the MMR with a peak latency of approximately 55 ms is the most human‐like rat MMR in the awake alert animal, as this component exhibits adaptation independent prediction error (Harms et al., 2014; Nakamura et al., 2011) and is substantially reduced with NMDAr antagonism (Harms et al., 2018). Furthermore, we and others have also found that the size of the negative MMR component at approximately 55 ms (referred as N54 component hereafter in this manuscript) scales in response to stimulus paradigm differences, where larger differences between the frequency (in Hz and perceived pitch) of the DEV and STD (Jalewa et al., 2021; Ruusuvirta et al., 2015; Shiramatsu et al., 2013), less probable DEV stimuli (Jalewa et al., 2021; Jung et al., 2013; Sivarao et al., 2014), and more temporally stable sound sequences individually induce larger MMRs in control rats (Astikainen et al., 2011; Jalewa et al., 2021). Taken together, these findings strengthen the notion that the rat brain can produce an MMR that shares many features with human MMN and that the N54 MMR component is the most human‐like, facilitating further work aimed at observing schizophrenia‐like MMR deficits in rat models of the disorder.

Based on epidemiological and genetic findings of schizophrenia, many animal models of schizophrenia risk factors have been developed and have been observed to exhibit a range of schizophrenia‐like features, including hypofunction of the NMDAr system. Although schizophrenia typically has an early adulthood onset, many of the risk factors for the disorder affect neural development, with key periods of vulnerability in the prenatal period, and in adolescence. Maternal immune activation (MIA) and early adolescent cannabinoid exposure (ACE) (Solowij & Michie, 2007) are two environmental factors known to increase 3‐ to 7‐fold (Brown & Derkits, 2010) and 2‐fold (Arseneault et al., 2004) risk of schizophrenia in offspring, respectively. Maternal infection during pregnancy is a significant risk factor for developing schizophrenia in the offspring later in life (Canetta & Brown, 2012) and this risk factor has been demonstrated in a number of in vivo models to be primarily due to the maternal immune response (Duchatel et al., 2016; Fatemi et al., 2005; Meehan et al., 2017; Meyer, 2013; Meyer et al., 2006; Murray et al., 2017; Rahman et al., 2017; Shi et al., 2003; Short et al., 2010; Weir et al., 2015; Zuckerman et al., 2003). A viral mimetic such as polyriboinosinic‐polyribocytidylic acid (Poly I:C, a double‐stranded synthetic RNA) is often used in rodent models to induce MIA. The gestational timing of the prenatal immune activation impacts behavioral, cognitive, and neurobiological outcomes in the offspring (Meyer et al., 2006, 2008). For instance, transient working memory impairments have been found in the offspring exposed to MIA at late gestation (gestational day 19, GD19) while prepulse inhibition deficits were found in males exposed at either early (GD10) or late gestation, but not in females (Meehan et al., 2017). Similarly, animal models of ACE induced by repeated administration of cannabinoid drugs, such as THC, or other CB1 agonists, are found to exhibit a range of behavioral alterations relevant to schizophrenia (Rubino & Parolaro, 2016), such as prepulse inhibition deficits (Llorente‐Berzal et al., 2011) and reduced social interaction (O'Shea et al., 2006). However, as schizophrenia in humans is not likely to be due to a single cause, animal models that utilize a combination of risk factors have the potential to more accurately model schizophrenia pathophysiology (for review, Dunn et al., 2020). Such investigations of two‐hit models have found that a combined exposure to MIA and ACE resulted in significant changes in the transcriptional networks involved in the neurotransmission, cellular signaling and schizophrenia (Hollins et al., 2016) along with sex‐specific effects in hypothalamic and microbiota abnormalities (Katz‐Barber et al., 2020).

While previous findings indicate that a range of neurobiological and even gastrointestinal outcomes can be induced by a two‐hit exposure to both MIA and ACE (Dunn et al., 2020; Hollins et al., 2016), these animals have not yet been examined on measures with face validity for their resemblance to schizophrenia electrophysiological phenotypes. Therefore, we set out to determine whether rats exposed to either MIA, ACE, or the two factors together, exhibit schizophrenia‐like impairments in MMRs to DEV stimuli using two different stimulus paradigms that affect precision weighting of the MMR signal in different ways. They are variations in deviance difference and variations in deviant probability. In the deviant difference condition, the probability of a deviant event is constant but the physical separation between the standard and deviant is altered. In this manipulation the higher precision weighting on the error signal (larger MMR) is thought to be due to the error falling further from the distribution defined by the model. In contrast, in the probability condition the physical difference is constant but the average number of repetitions between deviant sounds is altered. In this manipulation, the higher precision weighting on the error signal (larger MMR) is thought to be due primarily to the higher precision associated with the model for the deviating parameter (in this case, frequency^1^), when the deviant is rarer. These two manipulations were chosen because the disruptions to MMN in schizophrenia tend to be the largest when control MMN is at its largest, with probability and stimulus difference manipulations able to reveal these effects (Javitt et al., 1998). Both male and female animals were used to examine this relationship, as there are prominent sex differences in age of onset, course of illness, and severity of symptoms in schizophrenia (Gogos et al., 2020; Lins et al., 2019). In addition, prominent sex‐specific effects have been described in models of MIA (Carney, 2019; Gogos et al., 2020; Lins et al., 2019) and ACE (Borsoi et al., 2019; Lee et al., 2014; Miladinovic et al., 2020; Zamberletti et al., 2012), including two‐hit ACE models (Dunn et al., 2020). We (Harms et al., 2014; Jalewa et al., 2021) and others (Astikainen et al., 2011; Peter et al., 2010; Shiramatsu & Takahashi, 2018) have found that incremental/ascending frequency changes between standard and deviant of oddball sequences produce higher MMR amplitude in comparison to decremental/descending frequency changes. It has been speculated that this frequency‐dependent asymmetry is perhaps due to the natural environment of the rat being dominated by low‐frequency components so low‐frequency sounds are less salient (Shiramatsu & Takahashi, 2018). Because of this well‐known frequency‐dependent asymmetry of MMN amplitude in rats, we focused on the N54 MMR in response to high frequency deviants only. We hypothesize that rats exposed to both developmental risk factors (MIA and ACE) will exhibit maximal reduction in the amplitude of the N54 MMR component, the most human‐like MMR response.

## 2 METHOD

### 2.1 Ethics statement

All experiments were performed in accordance with the National Health and Medical Research Council's Australian code of practice for the care and use of animals for scientific purposes. The Animal Care and Ethics Committee, University of Newcastle, NSW, Australia approved the experimental protocols (Ethics approval number: A‐2016‐610). The surgical procedures were executed under maintained anesthesia and all efforts were made to reduce the number of animals and minimize the pain and suffering following surgery through the use of analgesics.

### 2.2 Breeding and development of two‐hit rat model

**FIGURE 1:**
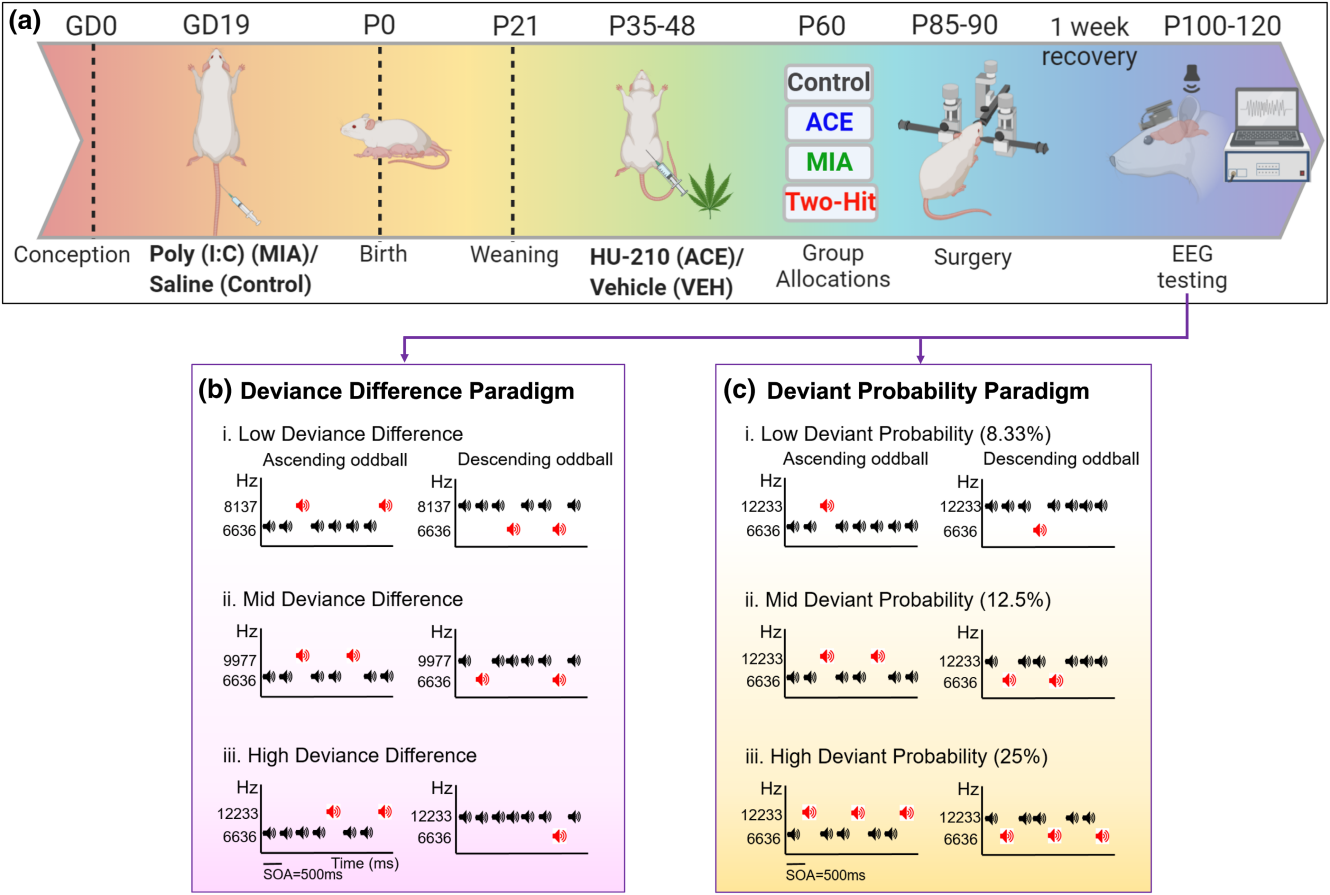
Experimental timeline. (a) Breeding and development of control, single‐hit, and two‐hit rats, followed by EEG testing after one week of post‐surgery recovery. Created with BioRender.com. (b) Deviance difference paradigm. Rats were presented with three conditions of increasing frequency difference between the standard (STD) and deviant (DEV) stimuli: i. Low (6636 Hz vs. 8137 Hz), ii. Mid (6636 Hz vs. 9977 Hz), and iii. High (6636 Hz vs. 12,233 Hz) with a 12.5% probability of occurrence of a DEV sound. (c) Deviant probability paradigm. Rats were presented with three different DEV probability conditions: i. low: 1/12 tones (8.33%), ii. Mid: 1/8 tones (12.5%), and iii. High: 1/4 tones (25%) with a high frequency difference between the STD (6636 Hz) and DEV (12,233 Hz). (b and c) The roles of STD and DEV in each condition reversed (a flip‐flop design), resulting in ascending (left) and descending (right) oddball sequences.

**FIGURE 2:**
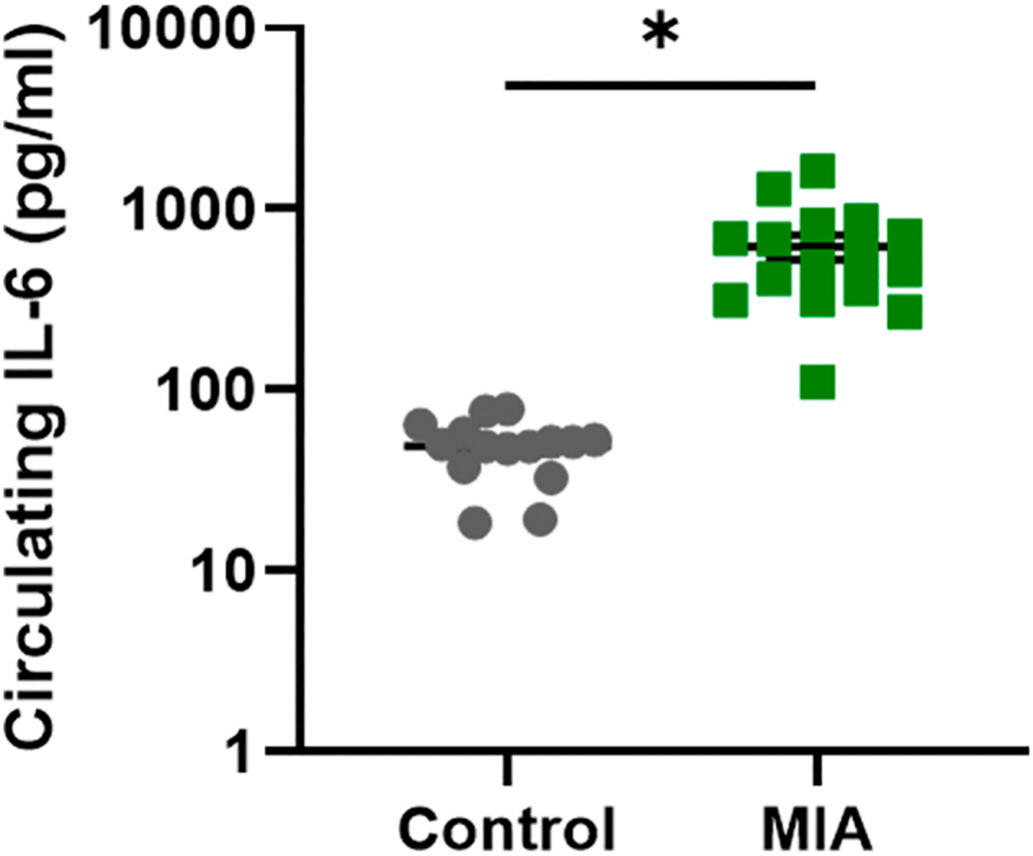
Dam plasma IL‐6 levels 2 hr post GD19 poly I:C administration. Graph shows log_10_ transformation ± SEM of circulating IL‐6 (pg/ml) in control (gray circles, *n* = 15) and MIA (green squares, *n* = 16) dams. Due to violation of the assumption of normality, plasma concentrations of IL‐6 in dams were analyzed using non‐parametric, independent samples Mann–Whitney U test (SPSS). The dams exposed to MIA on GD19 exhibited a significant increase (*p* < .001) in the levels of circulating IL‐6 relative to control dams. The MIA dam with the lowest concentration of IL‐6 of 110.641 pg/ml (lowermost green square) was 32.629 pg/ml higher than the control dam with the highest IL‐6 concentration (78.012 pg/ml).

Female rats in afternoon proestrous were time‐mated overnight and pregnancy was confirmed (gestational day 0, GD0) by sperm detection the next morning (see Figure 1 for a summary of the breeding timeline). On GD19, either saline (control group) or 5 mg/kg polyI:C (MIA group, Sigma Aldrich, Australia) was injected via the lateral tail vein under isoflurane anesthesia. IL‐6 assessment was performed to confirm MIA. After 2 hr of GD19 tail vein injection, blood was collected from the saphenous vein, centrifuged at 1400*g* for 15 min at 4°C and plasma was collected for IL‐6 assessment. Using a Quantikine IL‐6 ELISA kit (R&D systems Inc., USA), circulating levels of the pro‐inflammatory cytokine IL6 were quantified to confirm MIA after polyI:C administration (Figure 2). Offspring were weaned on postnatal day 22 (P22). HU‐210 (Sapphire Bioscience, Australia), a synthetic cannabinoid more potent and selective than the psychoactive constituent Δ9‐tetrahydrocannabinol (THC) (Martín‐Calderón et al., 1998; Ottani & Giuliani, 2001) of cannabis, was injected intraperitoneally once daily from P35 ‐ P48. Adolescent offspring were administered a dose of 0.1 mg/kg for males and 0.075 mg/kg for females (Lee et al., 2014; Wiley et al., 2017) dissolved in vehicle solution of Tween 80: DMSO: Saline in a 1: 1: 98 mixture (ACE group), or with vehicle alone (VEH group) (Figure 1a). Previous rat studies have used similar dosages of HU‐210 as administered in the current study. For instance, Ferrari et al. (2000) studied inhibitory effects of HU‐210 on male and female rat sexual behavior using escalating doses of HU‐210 including 0.025, 0.05, and 0.1 mg/kg body weight administered intraperitoneally in acute or subchronic regimes (once daily for 7 and 14 days). Out of all the doses of HU‐210, 0.1 mg/kg body weight dosage was found most effective leading to significant sexual impairments. Lee et al. (2014) investigated the effects of HU‐210 on adult rat hippocampal neurogenesis by administering the escalating doses of HU‐210 (0.025, 0.05, and 0.1 mg/kg), or vehicle during adolescence from postnatal day 35 to 46. Usage of similar dosage settings of HU‐210 by these studies and others (Farinha‐Ferreira et al., 2022; Lewis et al., 2012) suggest that the dose of HU‐210 administered in the current study is one that can certainly exert significant physiological effects.

Females were administered a lower dose of HU‐210 than males as female rats exhibited seizure behavior (unpublished observations) after 10–12 days when administered a HU‐210 dose of 0.1 mg/kg. Therefore, the dose was lowered to 0.075 mg/kg and no more seizures were observed. There are well‐known sex differences in cannabinoid pharmacology (Craft et al., 2013). In rodents, females have been found to be more sensitive than males to the effects of cannabinoids on tests of antinociception, motor activity, and reinforcing efficacy (Craft et al., 2013). One of the reasons underlying greater vulnerability of females to cannabinoids in comparison to males could be higher CB1 receptor density and function in males than females (Castelli et al., 2014). In general, studies on the effects of cannabinoid exposure during adolescence in both humans and rodents suggest that female adolescents are more likely than male adolescents to be deleteriously affected by cannabinoids (Craft et al., 2013). Recent studies on THC, which is much less potent than HU‐210, have also shown a markedly different THC metabolism in male and female adolescent rats (Ruiz et al., 2021), and female rats have been found to be more sensitive to the same dose of synthetic cannabinoids than male counterparts (Wiley et al., 2017). Only one rat/sex/adolescent group/litter was allocated to the experiments in order to minimize the litter effects. Subsequent surgeries, testing, and analysis were performed by researchers that were blind to MIA and ACE group.

### 2.3 Animals utilized in experiments

A total of 96 (48 male, 48 female) Wistar rats (age: 3–4 months) underwent surgeries for the study. There were eight experimental groups (*n* = 8–12 per group/sex) who were male and female offspring of the dams exposed to one of two treatments – either saline or maternal immune activation (MIA). Offspring of these dams were assigned at random to receive either vehicle or chronic cannabinoid exposure during adolescence. These treatments resulted in 4 groups of offspring – control, single hit of MIA alone, single hit of adolescent cannabinoid exposure (ACE) alone or two‐hit (MIA followed by ACE). All rats were housed in pairs in open‐top cages, in temperature‐ and humidity‐controlled conditions in the Behavioral Sciences animal facility at the University of Newcastle, under a 12 hr day‐night cycle (lights on at 07:00 hr), with ad libitum access to the food and water.

### 2.4 Mismatch response (MMR) recording

Surgery was performed to implant stainless steel screw electrodes on the skull of rats as previously published (Harms et al., 2014; Jalewa et al., 2021). Six 0.9 mm burr holes were drilled at locations corresponding to the four recording sites: two rostral sites 2.00 mm anterior to Bregma and 2.00 mm lateral to the midline, and two caudal sites 5.00 mm posterior to Bregma and 4.00 mm lateral to the midline, (Paxinos & Watson, 2006), the ground electrode (left posterior cortex, 2.00 mm anterior to Lambda and 2.50 mm left of the midline), and the reference electrode dorsal to the cerebellum (1.00 mm posterior to Lambda and 1.00 mm to the right of the midline). Testing was performed after a week of post‐surgery recovery. MMRs were investigated in awake, freely moving rats using a wireless‐10‐channel telemetric headstage (Multi Channel Systems, Germany) and acoustic stimuli were presented via a speaker in a sound attenuated chamber. Multi‐Channel Systems MCRack software was used to record the EEG data and digitization was performed at 2000 Hz (high pass filter 0.1 Hz, low pass filter 500 Hz, voltage range ±12.4 mV) (Harms et al., 2014; Jalewa et al., 2021).

### 2.5 Experiment design and stimuli

The rats utilized in this study were generated in five waves to ensure that rats could be tested within one month after surgery. Each wave was made up of offspring obtained from 8–12 dams with half of the dams exposed to saline and the other half administered Poly I:C on GD19 (described in Section 2.2). The order of presentation of the two stimulus paradigms, deviance difference and deviant probability, was the same for all the rats within a wave but was counter‐balanced across waves. For each rat, testing sessions of the two paradigms were at least 3 days apart. Within each paradigm (deviance difference and deviant probability) there were three different oddball sequences: low, mid, and high (Figure 1), which were presented as either ascending (high frequency DEV) or descending (low frequency DEV) sequences in a flip‐flop design (so that standard and deviant ERPs could be derived from tones with identical physical characteristics), totalling six different sequences. These sequences were presented in six different orders using a partial latin square design so that individual sequence types were not over‐represented at the beginning or end of the testing session (e.g. the two low probability sequences would not both be presented as the first two sequences together). Rats were allocated pseudo‐randomly to different sequence orders so that an even mix of sequence orders were used to generate data within each Treatment group. In the Deviance difference sequences, low (6636 and 8137 Hz), mid (6636 and 9977 Hz) and high (6636 and 12,233 Hz) frequency differences between the DEV and STD stimuli were employed (Figure 1b) with the roles of standard (87.5%) and deviant (12.5%) in each sequence reversing resulting in an ascending (low frequency standard and high frequency deviant) or a descending (high frequency standard and low frequency deviant) deviant sequence for each frequency difference (Jalewa et al., 2021). EEG was recorded for each rat on each of these six sequences (lasting 1 hr 33 min that included a minimum 1 min silence between each sequence). For each sequence type, a total of 200 deviants and 1400 standards were presented in a pseudorandom order with a stimulus onset asynchrony (SOA) of 500 ms, and no fewer than three standards between each deviant. In the Deviant Probability sequences, two fixed tones of 6636 Hz (a low frequency tone) and 12,233 Hz (a high frequency tone) were used with variable probabilities of occurrence of DEV [1/4 (25%, high), 1/8 (12.5%, mid) and1/12 (8.33%, low)] (Figure 1c). The sequences lasted longer than the deviance difference paradigm, to ensure a sufficient number of deviant stimuli presented for the low probability condition. Each session lasted 2 hr 13 min, and of a total of 2400 stimuli presented for each sequence type. There were 150, 300 and 600 deviants presented in the low, mid, and high probability conditions, respectively (Jalewa et al., 2021).

This design allowed us to examine the effect of treatment on MMRs elicited under the two different paradigms but also provided the opportunity to examine the effect of context induced by the different paradigms on MMRs generated by identical sequences except for whether sequence was presented as part of a probability manipulation of the deviant or a degree of deviance difference (from the standard). The two sequences that were identical were the High Deviance condition of the Deviance Difference sequences (Figure 1b iii) and the Mid‐Probability condition of the Deviant Probability sequences (Figure 1c ii): the deviant versus standard stimulus frequencies were 6636 Hz versus 12,233 Hz and the probability of the deviant was 12.5% in both cases. What varied across the two sequences was the context in which the data from the sequence was recorded – either as part of a range of deviance difference conditions or in the context of a range of deviant probability conditions.

The numbers of male and female animals assigned to each treatment and for whom data were available for each of the sequences are shown in Table 1. It is clear from this table that not all animals produced data for each sequence. Missing data were primarily due to deterioration of the headstage connector, excessive artifacts, or excessive movement from the rat, removing the headstage (see Section 2.6), either resulting in poor recordings, or recordings that yielded <100 trials per stimulus. Missing data were not a problem for comparisons of MMRs across the three probability conditions and across the three deviance difference conditions but were a problem when the MMRs from two identical stimulus conditions, described above, were compared across the probability paradigm and the deviance difference paradigm.

**TABLE 1:** The numbers of male and female animals assigned to each condition that produced data from the deviance difference sequences and deviant probability sequences

	Deviance difference sequences				Deviant probability sequences			
	Control	ACE	MIA	Two‐hit	Control	ACE	MIA	Two‐hit
Male	8	8	11	10	9	11	12	11
Female	10	9	11	9	11	9	10	11

Presentation software (Neurobehavioral Systems, Inc.) was used to generate 70 dB auditory stimuli of 100 ms duration with 10 ms rise/fall times. The stimuli were frequency‐modulated tones with carrier frequencies of 6636, 8137, 9977 and 12,233 Hz, modulated by 3% using frequencies of 98.1, 120.3, 147.5, and 180.8 Hz, respectively (Harms et al., 2014; Jalewa et al., 2021). These stimuli were used because use of pure tones at 8137 Hz resulted in a very large amplitude ERP, possibly because the 8 kHz tone was close to the natural resonant frequency of the holding chamber. Importantly, use of broadband stimuli removed the variation in the amplitude of ERPs to standard sounds of different frequencies.

### 2.6 Data extraction

Data were processed off‐line using EEGDisplay (version 6.4.1) (Harms et al., 2014; Jalewa et al., 2021). Channels with extreme artifacts (such as those caused by detachment of the wire to the recording electrode or excessive noise on a single channel) were identified by an observer blind to Treatment and excluded prior to further processing. The initial pre‐processing step involved exclusion of gross artifact intervals in the continuous EEG record using an automated algorithm that rejected signals exceeding 1400 μV. Thereafter, epochs were extracted from the continuous EEG consisting of 100 ms pre‐stimulus baseline and a 300 ms post‐stimulus interval. At epoch extraction, ERPs were baseline corrected over a 100 ms pre‐stimulus interval. Epochs were averaged for each rat and separately for each stimulus type. Only rats that had >100 trials for each stimulus type were included in further analyses. The latency ranges of individual ERP components were determined based on the morphologies of the ERPs, taken from grand averages and thereafter, their mean amplitude extracted for each rat. While multiple peaks were evident in the ERPs, only a negative component peaking at 54 ms, N54, was measured here as it is the most MMN like of the rat ERP peaks (Jalewa et al., 2021). Mean amplitude measures for N54 MMR component were extracted over latency windows 41.5–67.5 ms and 44–67 ms for the deviance difference experiment and the deviant probability experiment, respectively, using EEG Display (version 6.4.1). The extraction windows were based on inspection of the grand average waveforms over all animals for the two different paradigms. Because some animals had data missing for either one of the two paradigms, it should also be noted that some individual rats contributed data to both paradigms, and some to only one, therefore the extraction windows were slightly different for each experiment. As a flip‐flop control was employed, stimuli of the same frequency could be compared, e.g., 8137 Hz DEV versus 8137 Hz STD. The MMR was computed for each condition as the difference: DEV – STD for high‐frequency tones. While EEG was recorded over 4 electrodes (left/right, rostral/caudal) and measurements were conducted on each site separately, for all statistical analyses, mean MMR of measured amplitudes averaged over sites were used as the dependent variables for each animal.

### 2.7 Statistical analysis

Given female rats have been found to be more sensitive to synthetic cannabinoids than males (Craft et al., 2013; Lee et al., 2014) and because female rats showed adverse reactions to the 0.1 mg/kg dose in previous studies, in the current study, a lower dose of HU‐210 was administered to females compared to males. As this difference in the dose of HU‐210 may lead to variable effects of ACE on neurophysiology in males and females and may act as a confounding factor in the interpretation of sex differences, we performed separate analyses of male and female data.

The first analysis reported using IBM SPSS Statistics 26 software were two analyses of variance (ANOVAs) with independent groups on Treatment (4 levels – Control, MIA, ACE and Two‐Hit) and repeated measures (RM) on Sequence (3 levels for each paradigm – high, mid, low) conducted separately on the Deviance difference paradigm and the Deviant Probability paradigm with mean amplitudes of the N54 MMR component as the dependent variable. The second analysis was a Linear Mixed Model (LMM) analysis that examined context effects on MMRs derived from identical sequences in different contexts, LMM being necessary because of missing data for reasons outlined earlier. If significant main/interaction effects were observed suggesting treatment effects, two sample *t* tests comparing treatment with control without adjustment for multiple comparisons are reported, together with Hedge's *g* estimation of effect size of the treatment effect. To determine whether MMR reduction was driven by the change in responses to the DEV or the STD or both stimuli, we performed RM‐ANOVAs on DEV and STD ERPs separately.

## 3 RESULTS

### 3.1 Male but not female rats exhibit impact of treatment on the size of MMR under deviant probability manipulation

**FIGURE 3:**
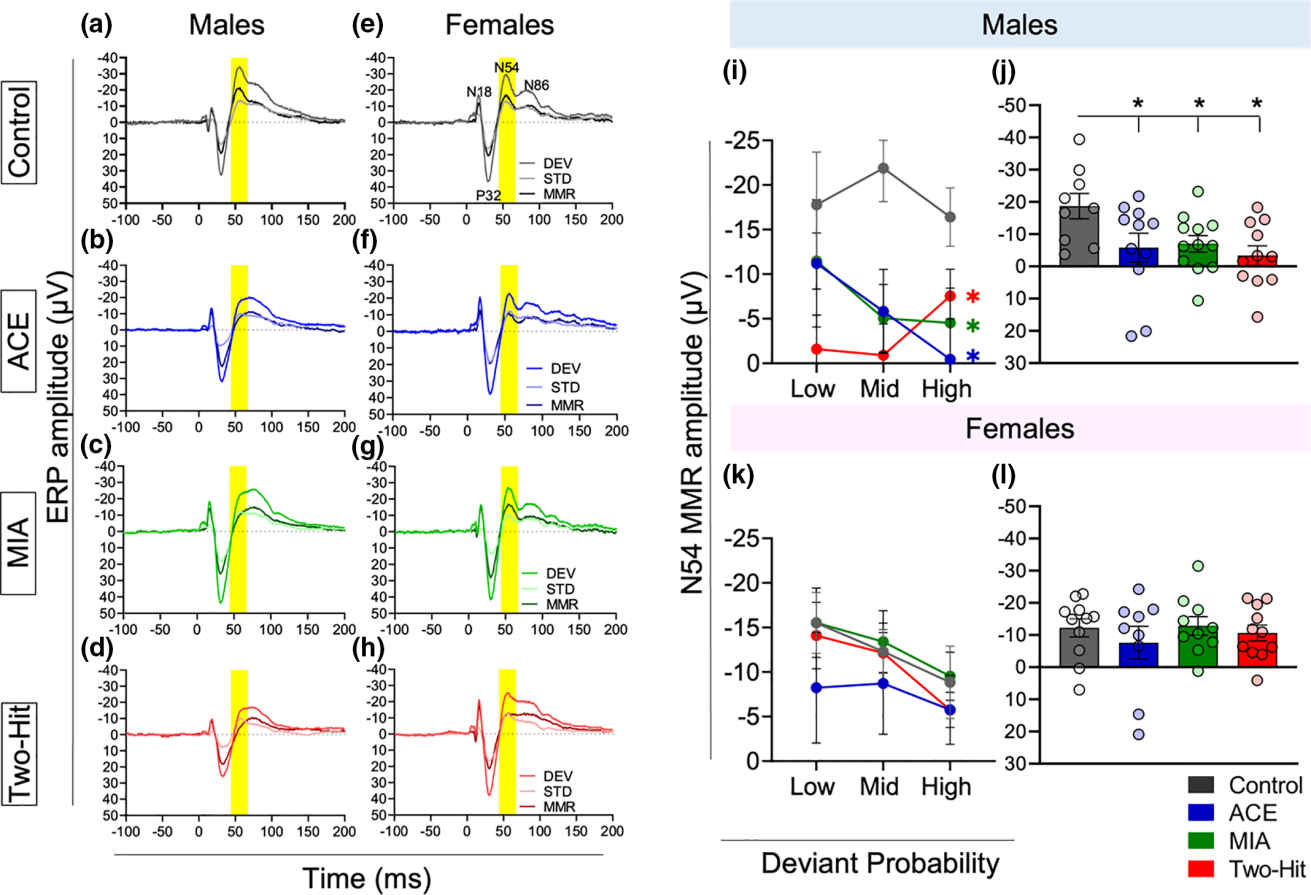
Effect of maternal immune activation (MIA) and adolescent cannabinoid exposure (ACE) on the mismatch responses (MMRs) under deviant probability manipulation. (a–h) grand averaged ERPs, averaged over low (8.33%), mid (12.5%), and high (25%) probabilities for all the experimental groups. The ERP plots show the response to DEV and STD stimuli along with the MMR difference waveform (DEV‐STD) (STD: 6636 Hz, DEV: 12,233 Hz) in control (a and e), ACE alone (b and f), MIA alone (c and g) and two‐hit (d and h) male rats (a–d) and female rats (e–h). Time window applied to extract the mean amplitude for the deviant probability experiment data set for the N54 component is shaded as gray. (i and j) Impact of treatment on MMR sensitivity to deviant probability manipulation in males. There was an impact of schizophrenia risk factors on the MMR mean amplitude (± standard error) of N54 as revealed by pairwise comparisons. There were no outliers identified in the male data and equality of variance across groups was not violated. (k and l) impact of treatment on MMR sensitivity to deviant probability manipulation in females. There were no treatment effects (*p* > .05) on the N54 MMR size. One animal from the MIA group produced an outlier, large negative amplitudes, in the medium and low probability conditions. Equality of variance across groups was not violated.

**FIGURE 4:**
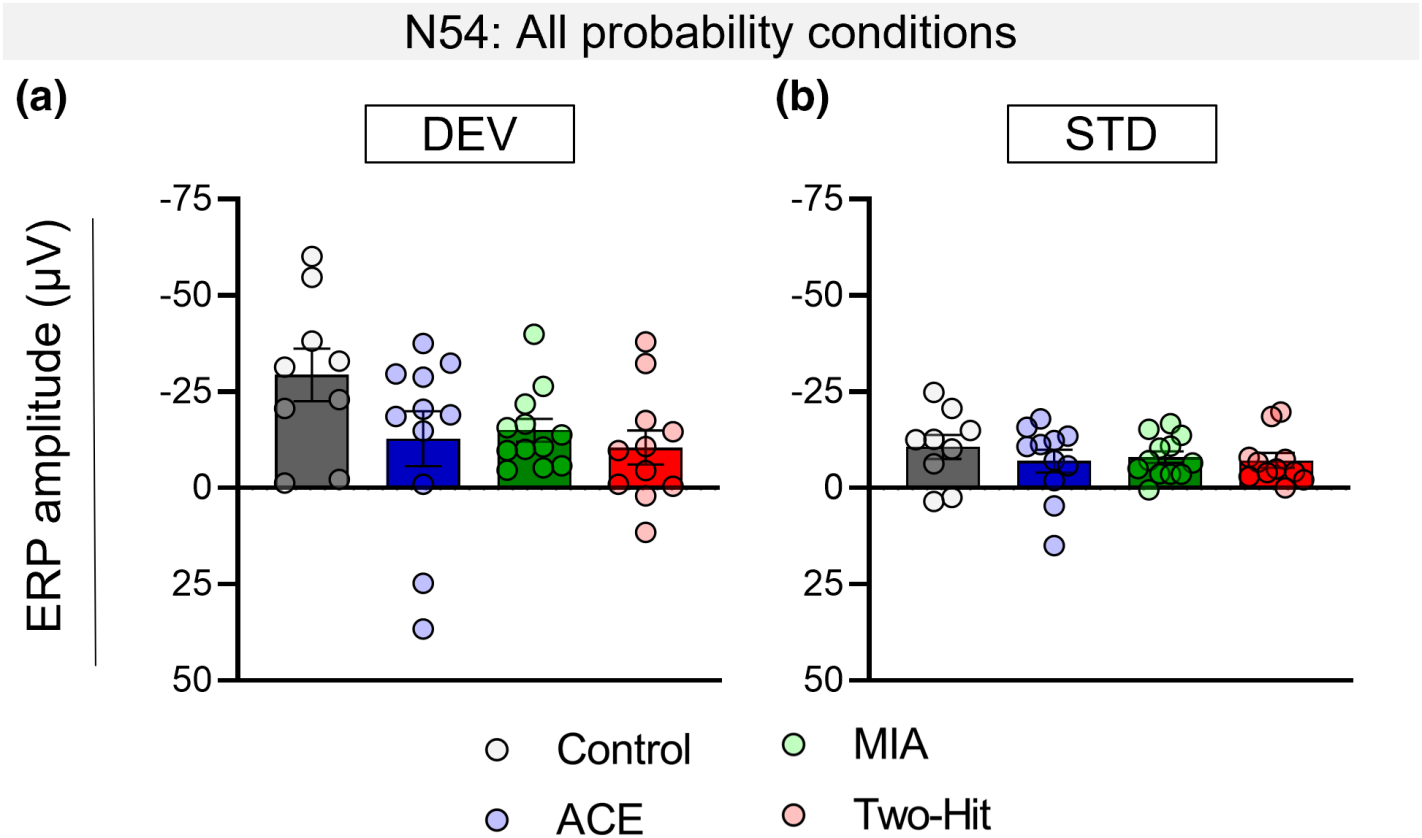
Altered response to deviant (DEV) but not standard (STD) stimuli drives the MMR reduction in ACE, MIA, and two‐hit rats. Responses to DEV and STD stimuli averaged over low (8.33%), mid (12.5%), and high (25%) probabilities in male rats. Mean amplitudes (±standard error, SE) of N54 component in response to the DEV stimulus (a) and STD stimulus (b).

In male rats, the treatments reduced the overall amplitude of the N54 MMR,^2^ in both the single‐hit (ACE alone and MIA alone) and two‐hit groups, relative to the control group (main effect of Treatment: *F*_[3,39]_ = 3.340, *p* = .029, *η* ^*2*^ = 0.204, Figure 3a–d), as revealed by post hoc pairwise comparisons (Control vs. ACE, *t*[18] = 2.115, *p* = .049, Hedge's *g* = 0.911; Control vs. MIA, *t*[19] = 2.597, *p* = .018, Hedge's *g* = 1.099; Control vs. Two‐hit, *t*[18] = 3.137, *p* = .006, Hedge's *g* = 1.35, Figure 3i). There was no Probability main effect, nor a Probability × Treatment interaction in males (*p* > .05). We found that N54 MMR reduction in the treatment groups was driven by the reduced response to DEV stimuli (Stimulus × Treatment interaction effect: *F*[3,39] = 3.340, *p* = .029, *η* ^2^ = .204),^3^ with pairwise comparisons confirming significant reduction in the response to DEV stimuli in MIA (t[19] = 2.119, *p* = .047; Hedge's *g* = 0.897), and two‐hit (t[18] = 2.405, *p* = .027; Hedge's *g* = 1.035) males, relative to controls (Figure 4a), but the reduction in ACE animals not reaching significance (t[18] = 1.650, *p* = .116, Hedge's *g* = 0.710). The response to STD stimuli was not altered (All Control vs. Treatment group comparison *p*‐values >.05; Figure 4b).

In females in contrast, there was a significant effect of Probability on MMR N54 amplitude (*F*_[2,72]_ = 7.218, *p* = .002, *η* ^*2*^ = 0.163), but neither Treatment,^4^ nor its interaction with Probability, had a significant impact on the MMR size (*p* > .05, Figure 3e–h,k).

### 3.2 No significant treatment effect on MMR to degree of deviance difference

**FIGURE 5:**
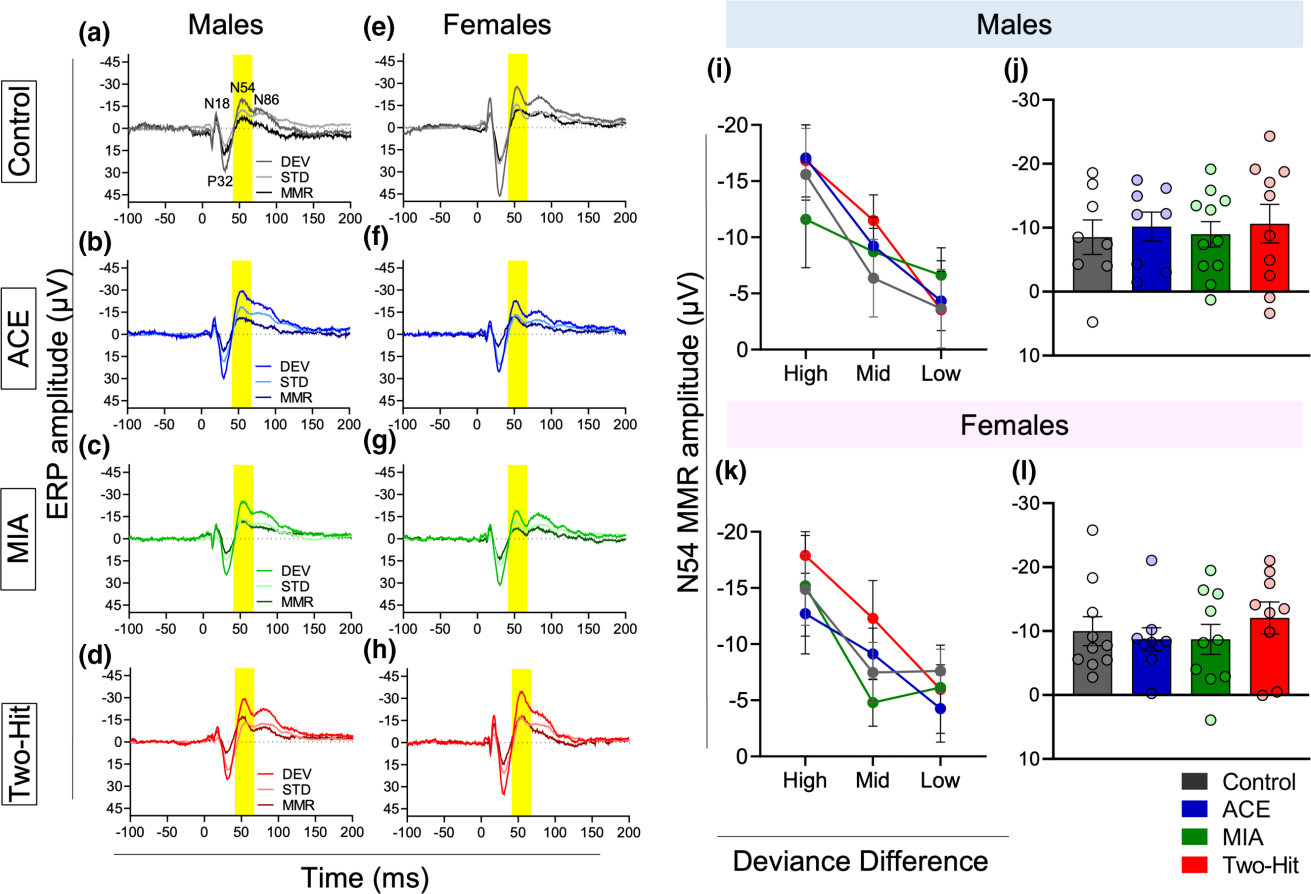
Effect of maternal immune activation (MIA) and adolescent cannabinoid exposure (ACE) on the mismatch responses (MMRs) under multiple deviance differences. (a–h) Grand averaged ERPs for all the experimental groups. The ERP plots show the response to DEV and STD stimuli along with the MMR difference waveform (DEV‐STD) averaged for the deviance difference conditions in control (a and e), ACE alone (b and f), MIA alone (c and g), and two‐hit (d and h) male rats (a–d), and female rats (e–h). Time window applied to extract the mean amplitudes for the deviance difference experiment dataset for the N54 component is shaded gray. (i and j) impact of treatment on MMR sensitivity to deviance difference manipulation in males. The graph shows unaffected N54 MMR amplitude (±standard error, SE) in treatment groups when compared to control group. (k and l) Impact of treatment on MMR sensitivity to deviance difference manipulation in females. The graph shows absence of significant treatment effect on N54 MMR amplitude (±standard error, SE).

The effect of degree of frequency difference between DEV and STD on MMR component amplitudes is shown in Figure 5 for each treatment group. It is clear that the negative component amplitudes tended to decrease when the standard and deviant were closer in frequency but the N54 response was not differently affected in control versus treatment groups. As expected, the N54 MMR differed in accordance with deviance difference in both male (*F*_[2,66]_ = 15.948, *p* < .001, *η* ^2^ = .326) and female (*F*_[2,68]_ = 14.487, *p* < .001, *η* ^2^ = .299) rats, with larger frequency differences between the STD and DEV stimuli producing the largest MMR (Figure 5i,j).

Neither male nor female rats exhibited overall effects of treatment on the N54 MMR component (Treatment main effects: males *F*_[3,33]_ = 0.152, *p* = .982, *η* ^2^ = 0.014; females *F*_[3,34]_ = 0.472, *p* = .703, *η* ^2^ = .040). For neither sex, did treatment affect MMR amplitude changes to the degree of deviance difference (Deviance × Treatment interaction: males *F*_[6,66]_ = 0.754, *p* = .609, *η* ^2^ = .064, Figure 5i; females: *F*_[6,68]_ = 0.699, *p* = .652, *η* ^2^ = .058, Figure 5j).

### 3.3 Context‐dependence of treatment effects on MMR

As noted earlier (Section 2.5), the design of the study provided the opportunity to examine the effect of context induced by the different paradigms (manipulation of the Probability of the Deviant or degree of Deviance Difference) on MMRs to identical sequences: the High Deviance condition of the Deviance Difference manipulations (Figure 1b iii) and the Mid‐Probability condition of the Deviant Probability manipulations (Figure 1c ii). A post‐hoc examination of the context sensitivity of the treatment effects on MMR was conducted by comparing N54 as a function of Treatment in males and females separately. A linear mixed models (LMM) approach determined the effect of treatment, context and treatment by context interaction on N54 amplitude. The LMM approach was chosen due to the propensity for missing data across contexts Loss of data was due to deterioration of the electrode connector over time, given that the data from the stimulus conditions (deviance difference, probability) were collected over a 3–4 week period. The LMM analysis uses all available information but assumes data were missing at random over the two contexts. Little's (1988) missing completely at random test revealed that this assumption was not violated for either the male or female rats (*χ* ^2^ = 1.96, *p* = .374 and *χ* ^2^ = 3.343, *p* = .188, respectively). This is consistent with the design of the study where the order of stimulus conditions across sessions/days was counterbalanced across animals. Correlation between N54 amplitudes in the two contexts due to repeated measures was modeled using a residual covariance matrix with unstructured form and with maximum likelihood estimation.

**FIGURE 6:**
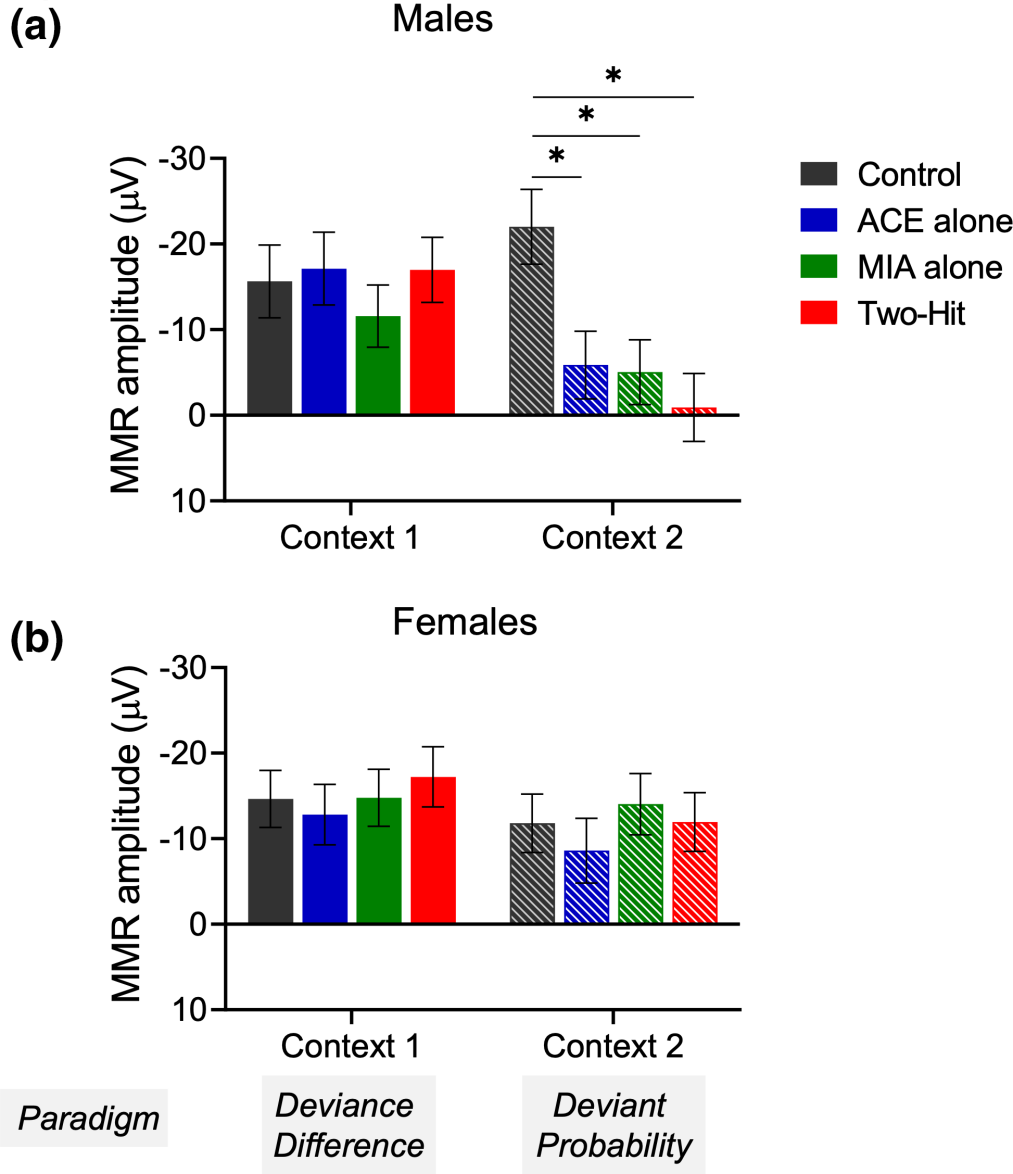
Male and female mean N54 amplitudes to identical oddball sequences in a deviance difference context and in a probability context. A separate sub‐analysis for male (a) and female (b) rats using a linear mixed models (LMM) approach of the two identical sequences but in different contexts replicates the results from the fuller analysis but provides a stronger test of context sensitivity of treatment effects as the two stimulus sequences are identical.

LMM analysis of the male data revealed significant main effects of Treatment (*F*_[3,58.9]_ = 3.057, *p* = .035), Context (*F*_[1,60.7]_ = 6.428, *p* = .014) and the interaction between Treatment and Context (*F*_[3,60.4]_ = 2.95, *p* = .040). LSD comparisons of the three Treatment N54 mean MMR amplitudes with Control revealed that none of these comparisons were significant for the deviant difference context (*p* range from .445 to .805) but all were significant for the deviant probability context (Control vs. ACE, *p* = .006; Control vs. MIA, *p* = .004; and Control vs. two‐hit, *p* = .001, respectively, Figure 6). For each comparison, the Control N54 mean MMR amplitude was significantly larger than the Treatment N54 mean amplitude in the probability context.

The equivalent analysis of the female data revealed no significant main effects, nor a significant interaction (*p* values range from .168–.908).

In summary, Treatment effects on MMR amplitude are sensitive to the stimulus context in male rats only. Female rats do not show a similar sensitivity, nor do they exhibit any significant effects of Treatment.

## 4 DISCUSSION

We investigated MMRs measured from the N54 component, the most human MMN‐like component in rats (Jalewa et al., 2021), in awake freely moving male and female rats to study the effect of cumulative schizophrenia risk exposures on the rat brain's predictive coding system in two different stimulus conditions. Clearly, the treatments based on schizophrenia risk factors did not produce robust reductions in the N54 MMR component in general. The treatment effects that were observed were condition‐dependent and possibly sex‐dependent (see caveats below). A significant reduction in N54 MMR amplitude only occurred when deviant probability was manipulated, and only in male rats.

Our prediction that the most human‐like of the rat MMR responses would be reduced maximally in those animals exposed to both developmental risk factors (MIA and ACE) was not supported across stimulus conditions. However, ACE alone, MIA alone and the combined effect of MIA and ACE produced significant N54 MMR amplitude reductions with large effect size overall (*η* ^*2*^ of 0.204) in the three male treatment groups relative to the control group. ACE and MIA therefore appear to have impacted a process that contributes to MMR amplitudes under a specific condition only. The results therefore do not resemble what we might expect if the treatments had induced a generalized NMDAr dysfunction leading to less differentiation between responses to STD and DEV tones as seen in schizophrenia. It is important to note in interpreting the results of this study that the experimental design ensured that (i) the order in which the two manipulations (degree of deviance difference and deviant probability) were delivered was counterbalanced across successive waves of rats and (ii) the order in which the three oddball sequences within each manipulation condition and their flip‐flop counterparts were presented to each rat was allocated evenly across treatment groups so that no treatment group would be over‐represented by animals that were exposed to the sequence in a specific order. Given the even allocation of sequence orders conducted in this experiment, it is highly unlikely that the treatment effects in the probability condition have occurred by chance or were due to systematic differences in the order in which stimulus conditions were presented. Understanding why this condition might be more susceptible to the risk factor manipulations therefore requires a careful consideration of what is different about the environments created in the two conditions.

It is not immediately clear why the treatment exposures preferentially affect MMRs in one condition (deviant probability), and not the other (deviance difference). Due to the tonotopic organization of the auditory system, the modulation of MMR amplitude to different levels of deviant frequency will be influenced by a spatial differential in the region of activated cortex. Representing the degree of error in predictions in this sense will presumably be informed by this spatial identifier (i.e., how distinctive the representational fields are, Näätänen & Alho, 1997). In contrast, the responsive locations to deviants and standards within the probability manipulations do not change. The expression of probability differences between the standard and deviant is arguably more reliant on an accumulation of information over time to weight relative likelihoods of the two frequencies. For the probability condition session, each of the sequences the rats heard contained a different number of deviating events and therefore a difference in the average number of standard repeats between deviant events. Theoretically, the different deviant probabilities should modulate the potential precision of the predictive model, with precision inversely proportionate to deviant probability (Garrido et al., 2008). The male control rats did not show the expected precision‐weighting based on within‐sequence probability differences suggesting that even control males were insensitive to deviant probability effects, but in addition, the overall precision‐weighting on the deviant response was significantly lower in the male treatment groups regardless of probability. The lack of an expected precision‐weighting in untreated males may be a reflection of the increased risk for schizophrenia in males (Iacono & Beiser, 1992), and being a male may be its own ‘single‐hit’ risk factor in itself. Such effects could be hormone‐driven, with experiments in mice observing that supplementation with estradiol reversed schizophrenia‐like EEG changes (disrupted high‐frequency oscillations) in ovariectomised female mice (Schroeder et al., 2017). The overall smaller MMR under these conditions suggests that male treatment rats are particularly sensitive to this form of volatility (i.e., the rate of model deviations), but the absence of treatment effects in other conditions (and in female rats generally) indicates that this is not likely to be due to any generalized change in the mechanisms of MMR generation.

The importance of the context induced by the probability manipulation is further indicated by the post‐hoc analysis of MMR amplitudes to identical sequences across the two testing days. The MMR in treatment rats to a 12,233 Hz deviant among 6636 Hz standards was significantly smaller in the probability manipulation than the deviance manipulation, and only differed significantly from controls in the probability manipulation experiment. Given that these sequences were identical, the result implies that treatment rats *could* produce an equivalent precision‐weighting on this deviant response but *did not* do so in an experimental session where probability was manipulated. Additional analysis of DEV and STD amplitudes revealed that the treatment‐induced N54 MMR attenuation was driven by a reduced response to DEV stimuli and not a difference in STDs (Figure 4a). This finding suggests that schizophrenia risk factors influenced the prediction error response within this condition and not adaptation, which is consistent with recent findings in the schizophrenia literature (Koshiyama et al., 2020). Overall, the absence of adjustment in the precision‐weighting in male rats suggests that the models were insensitive to the local probabilistic information. The overall smaller MMR under these conditions suggests that male treatment rats are particularly sensitive to this form of volatility (i.e., the rate of model deviations) but the absence of treatment effects in other conditions (and in female rats generally) indicates that this is not likely to be due to any fundamental change in the mechanisms of MMR generation.

Interpretation of sex differences in these data is challenging particularly with respect to the ACE and two‐hit groups. It is acknowledged that there are caveats to the interpretation of sex differences because male and female rats were not compared directly, so an inference regarding male–female differences cannot be drawn from these data. Male rats were administered a higher dose of the cannabinoid HU‐210 due to male rodents being less sensitive to the pharmacological effects of synthetic cannabinoids compared to females (Craft et al., 2013; Lee et al., 2014; Wiley et al., 2017). This dosage difference complicates a determination of whether the effect in males is due to the higher cannabinoid dose or genuine sex differences. However, this caveat does not apply to the MIA alone treatment where the group of male offspring also exhibited a reduced N54 MMR amplitude relative to the Control group. Further support for sex‐differences in the probability condition can be seen in the overall insensitivity of male offspring to altered DEV probabilities in contrast to females. This observation is also in contrast to the clear sensitivity to degree of DEV difference where both male and female offspring exhibited the expected effects. As MIA and ACE showed comparable effects on the precision weighted MMR signal, both MIA and ACE could be acting on a final common pathway, perhaps each perturbing the system to its limit, producing floor effects, such that no additional impact can be observed after the addition of the second hit.

Previously, we observed increased expression of the NMDAR subunit, NR2A, in the auditory cortex (believed to be one of the regions involved in MMR generation) following MIA, and this increase drove an increase in the NR2A:2B ratio (Rahman et al., 2017). NR2B confers greater activity to the NMDAR, thus increased 2A:2B may suggest reduced NMDAR activity. Rubino et al. (2015) also found that ACE altered the maturational fluctuations of NMDA subunits during adolescence, leading to higher amounts of prefrontal NR2A during adolescence in female rats. While NMDAR expression profiles in the current model have yet to be published, our findings in the current study indicate that MIA, ACE or both together, may be used to examine schizophrenia‐like MMR reductions in future studies: first, to explore the neurobiology underlying such effects (i.e. which brain regions/cell types are involved), and second, to examine whether potential new therapeutics for schizophrenia can increase MMRs.

While the present results cannot speak directly to the molecular underpinnings of MMR deficits observed exclusively in male rats exposed to MIA, ACE or both, and would certainly benefit from an investigation of molecular mechanisms, we speculate the differences could be due to the different ACE doses used in this study along with the sex‐specific differences in the NMDA receptor system known to play a role in the generation of primate MMN (Gil‐da‐Costa et al., 2013), and rodent MMRs (Schuelert et al., 2018; Tikhonravov et al., 2008). One of the key candidate hypotheses for the pathophysiology of schizophrenia is dysfunction in neurotransmission via NMDA‐type glutamate receptors (Kantrowitz & Javitt, 2010) that affects many pathways and systems, including auditory pathway functioning. Evidence such as the inhibition of MMN generation by NMDA antagonists (Javitt et al., 1996; Tikhonravov et al., 2008) suggests a role of NMDAR system in the generation of MMN (Tikhonravov et al., 2008; Todd et al., 2013). Sex differences in the glutamate system have been well reported, with an overall increase in glutamate transmission in females compared to males in both animal and human studies (Wickens et al., 2018). This suggest that higher levels of both NR1 and NR2B NMDA subunits and increased NMDAR sensitivity to glutamate in females may be protective and lead to differences in schizophrenia symptomology (Wickens et al., 2018). Moreover, studies have reported sex differences in glutamate transmission in MIA and ACE rodent models. Rahman et al. (2017) found exaggerated changes in NMDAR in male offspring compared to females, with increased NR2A binding in the striatum and cortex and elevated mRNA levels of NR2A in MIA males but not females, indicating that MIA may alter glutamatergic signaling in the cortical regions via alterations in NMDAR indices, but in a sex‐dependent manner. These findings suggest that sex‐specific alterations in the NMDAR system may underlie the sex‐specific effects observed in this study. Unfortunately, there is a dearth of studies considering sex differences in MMN in humans as well as rodents, and more research is required to understand such differences to utilize the translatable potential of MMN findings in schizophrenia.

In summary, it is clear that rat MMRs to deviants in certain oddball sequences (such as variations in deviant/standard difference) are robust to quite substantial perturbations of the developing brain, such as those induced by maternal infection or chronic use of cannabis during adolescence. The effects that we have observed on offspring subjected to the developmental risk factors that we examined are rather subtle and only evident in one particular context of those contexts investigated. However, the results are nonetheless informative and intriguing. Firstly, they are the first observations of MMR reductions in an animal model of schizophrenia based on endogenous factors and not due to acute pharmacological manipulations. For instance, rodents exposed to acute NMDAr antagonists such as MK‐801 exhibit reduced MMR (Harms et al., 2020). However, individuals with schizophrenia have an endogenous brain state that consistently produces reduced MMN and is likely more complex than systemic and acute NMDAr hypofunction that affects all cells and regions. Our findings show that rodent MMR is reduced in response to schizophrenia risk factors in a paradigm that manipulates factors shown to affect MMN reduction in schizophrenia (Javitt et al., 1998; Shelley et al., 1999). Secondly, an insensitivity to probability manipulation has been seen in schizophrenia where patients do not show the expected increase in MMN with lowered deviant probability (Javitt et al., 1998). However, meta‐analyses do not support deviant probability to be an important influence on the effect size for group differences (Erickson et al., 2016). It may be that in schizophrenia, like in our treated male rodents, the context of experiencing the manipulations of probability in the same testing session is important to exposing this general decrease in weighting of MMR with this form of precision modulation. Finally, determining how contextual sensitivity to probability is expressed becomes an important focus of future work on both schizophrenia risk as well as the basic mechanisms of MMN/R.

In conclusion, the results show the potential of MMN in a preclinical developmental risk factor model for schizophrenia as a way forward to explore the neurobiology of such effects, improve the reliability of schizophrenia rodent model validation, and to test novel pro‐cognitive therapeutics. Ideally the present results require replication and could be used as a basis for more targeted investigation of both the sex and context specificity of the treatment impact on MMR in rodents. The results also serve as another example of the complexity of the neurobiological and neurophysiological system that supports the perceptual inferences expressed in MMN.

## AUTHOR CONTRIBUTIONS

**Jaishree Jalewa:** Conceptualization; data curation; formal analysis; investigation; methodology; validation; visualization; writing – original draft; writing – review and editing. **Juanita Todd:** Conceptualization; data curation; formal analysis; investigation; methodology; supervision; validation; visualization; writing – review and editing. **Patricia T. Michie:** Conceptualization; data curation; formal analysis; investigation; methodology; supervision; validation; visualization; writing – review and editing. **Deborah Hodgson:** Conceptualization; funding acquisition; investigation; resources; supervision; writing – review and editing. **Lauren Harms:** Conceptualization; data curation; formal analysis; funding acquisition; investigation; methodology; project administration; resources; software; supervision; validation; visualization; writing – review and editing.

## FUNDING INFORMATION

This work was supported by the Australian National Health and Medical Research Council (Grant No. APP1109283) to the Hodgson laboratory. JJ was supported by a University of Newcastle Postgraduate Research Scholarship, funded by the Australian government's Research Training Program.

## CONFLICT OF INTEREST

The authors report no financial interests or potential conflicts of interest.

## Data Availability

Data available on request from the authors.

## Footnotes

^1^
Frequency rather than duration as a deviant attribute was selected because there are little published data on duration discrimination in rodents (but see Pai et al., 2011) to guide the choice of duration differences in a degree of deviance manipulation. In contrast there are a number of reports of frequency discrimination in rats (Kelly & Masterton, 1977; Sloan et al., 2009; Talwar & Gerstein, 1998). It is recognized that duration deviants seem to be more sensitive than frequency deviants in revealing an MMN impairment in schizophrenia, particularly in the early stages of the illness (Todd et al., 2008) but a meta‐analysis found that frequency deviants produce a substantial effect size of 0.72 (Erickson et al., 2016) for patient vs. healthy control comparisons.

^2^
There was no effect of treatment on other components: N18, P32, and N86.

^3^
Note that the Stimulus × Treatment interaction in this analysis is equivalent to a test of the main effect of treatment in the analysis of the MMR, the difference between DEV and STD.

^4^
There were no treatment effects on other components either: N18, P32 and N86.
